# The safety of high dose continuous propofol infusion in the pediatric intensive care unit at an academic medical center

**DOI:** 10.3389/fped.2025.1718328

**Published:** 2026-01-12

**Authors:** Oluwatomini A. Fashina, Grace M. Arteaga, Sheri S. Crow, Rahul Kashyap, Ryan D. Frank, Xai Khang, Nicole M. Andrijasevic, Danette L. Bruns, Ashley V. Wong Grossman

**Affiliations:** 1 Department of Pediatrics, Mayo Clinic Children’s, Rochester, MN, United States; 2 Department of Pediatrics, Division of Pediatric Critical Care Medicine, Mayo Clinic Children’s, Rochester, MN, United States; 3 Department of Anesthesiology and Perioperative Medicine, Mayo Clinic, Rochester, MN, United States; 4 Division of Clinical Trials and Biostatistics, Mayo Clinic, Rochester, MN, United States; 5 Department of Anesthesiology and Perioperative Medicine, Anesthesia Clinical Research Unit, Mayo Clinic Children’s, Rochester, MN, United States

**Keywords:** analgesia, pediatric intensive care unit, pediatrics, propofol, sedation

## Abstract

**Introduction:**

Adequate sedation is crucial in the care of critically ill children. Propofol, known for its rapid onset, reliable patient response, and short duration of action, offers an attractive pharmacologic profile. However, concerns for propofol-related infusion syndrome (PRIS) have limited its use in pediatric critical care. This study examined the relationship between propofol administration and PRIS characteristics in a single medical-surgical pediatric intensive care unit (PICU). We hypothesized that continuous propofol infusion, even when judiciously exceeding current recommended doses and durations, is not associated with cardiovascular collapse, metabolic acidosis, or other PRIS-related characteristics.

**Methods:**

This is a retrospective single-center study in the non-cardiac medical/surgical PICU at a tertiary academic medical center of 554 patients. Children aged 0–17 years admitted to the PICU from 1/1/2000 to 9/30/2024, who received propofol infusions for ≥12 h and/or ≥2.4 mg/kg/hour (40 mcg/kg/min) were included. The primary outcome was in-hospital mortality; secondary outcomes included intensive care unit (ICU) length of stay, bradycardia during infusion, and metabolic acidosis during infusion. Data was compared across age groups (<6 months, 6–12 months, 1–5 years, 6–17 years) and medical/surgical diagnosis using the chi-square test for homogeneity and the Kruskal–Wallis tests.

**Results:**

Among 554 patients, the median age was 5.5 (IQR 1.5–12.1) years. The median propofol infusion rate was 3.0 (IQR 1.7–4.2) mg/kg/hour [50 (IQR 28–70) mcg/kg/min], median duration was 22.8 (IQR 16.4–42.4) hours, and median ICU length of stay was 4.7 (IQR 2.0–10.4) days. One case of PRIS was observed (0.2%). There were 26 (4.7%) in-hospital deaths, nonattributable to PRIS. Bradycardia occurred in 146 (26.4%) patients, and 19 (3.4%) patients developed metabolic acidosis.

**Discussion:**

In this single-center retrospective study, one case of PRIS was identified. While rare, its occurrence underscores the need for vigilance. Propofol remains a viable sedation option when used with appropriate monitoring in pediatric critical care.

## Introduction

Adequate sedation and anesthesia are core components in the care of critically ill children. Propofol offers a favorable pharmacologic profile with a rapid onset, consistent response, and short duration of action. However, early enthusiasm diminished as case reports emerged in the late 1990s linking propofol administration to metabolic acidosis, rhabdomyolysis, cardiovascular collapse, and death. This constellation of symptoms was subsequently defined as propofol-related infusion syndrome (PRIS) in 1998 (1). Reported risk factors include higher doses (>4 mg/kg/hour or 67 mcg/kg/min), prolonged durations (>48 h), younger ages (<12 months), critical illness and concomitant catecholamine and/or corticosteroid use (1–3). Further, the official Federal Drug Administration (FDA) label for Propofol (Diprivan) included a specific warning related to the use of propofol for continuous sedation in the pediatric population, stating that propofol is not approved for use in pediatric patients for sedation in the ICU, initially published in 1991 and revised on August of 2022, and that there is a risk of fatal adverse events in this population (4). In the critical care setting, clinical features of critical illness—such as bradycardia, metabolic acidosis, and cardiovascular collapse—are often indistinguishable from propofol-related adverse events, which may contribute to skepticism regarding whether the observed phenomena are related to propofol or the underlying critical illness (3).

Despite initial concern for PRIS, propofol administration in pediatric critical care continues to rise. A recent survey of pediatric intensivists reported that nearly 80% of respondents utilize propofol sedation in the Pediatric Intensive Care Unit (PICU) (5, 6). This widespread use has renewed interest in re-evaluating propofol-related adverse events in the modern era. Initial reviews suggest that PRIS may be rarer than originally reported and suggest that with lower doses (≤4 mg/kg/hour or 67 mcg/kg/min) and shorter durations (≤48 h), propofol administration in the PICU can be accomplished without adverse events (7–9). However, small sample sizes, variation in PRIS definition, and failure to account for pre-ICU health status, challenge the interpretation of these results. In addition, nearly all prior investigations of propofol in the PICU have included a substantial portion of children status post cardiopulmonary bypass surgery (CPB) (5–7). CPB is a well-established risk factor for hemodynamic instability, myocardial dysfunction, and lactic acidosis during the ICU period. The inclusion of post-cardiac surgical patients in these studies makes it difficult to discern whether PRIS-related characteristics are attributable to propofol itself or as a consequence of CPB-related hemodynamic and metabolic derangements. Finally, survey research suggests that modern propofol use often exceeds the recommended dose and duration ranges reported in the literature (6, 7). To date, clinical experience with higher-dose, prolonged-duration propofol administration has yet to be evaluated for adverse events and PRIS-related findings in a non-cardiac PICU population.

To address this knowledge gap, we conducted a retrospective review of the clinical course of all patients receiving propofol infusions during admission to a medical/surgical PICU. Our objective was to evaluate the relationship between propofol administration and PRIS-related characteristics. We hypothesized that continuous propofol infusion would not be associated with cardiovascular collapse, metabolic acidosis, or other PRIS-characteristics, even when administered at higher doses and longer durations than currently recommended.

## Methods

### Ethics

The study, titled “The Use of Continuous Propofol in a Pediatric Intensive Care Unit”, was approved by the Mayo Foundation for Medical Education and Research Institutional Review Board (IRB Number: 16-00346) on 24 October 2016. The study was conducted in accordance with the ethical standards outlined in the 1964 Declaration of Helsinki and its subsequent amendments. The legal guardians of all included patients had provided prior written consent for research participation, in accordance with Minnesota state law.

### Study design and setting

We performed a retrospective chart review of all patients admitted to the medical/surgical PICU at Mayo Clinic Children's between 1 January 2000 and 30 September 2024. The 20-bed PICU is a Level 1 trauma center admitting medical and surgical pediatric patients, including solid-organ transplant recipients, except for immediate post-heart transplant patients, who are cared for in a separate cardiovascular ICU.

### Inclusion and exclusion criteria

**Figure 1:**
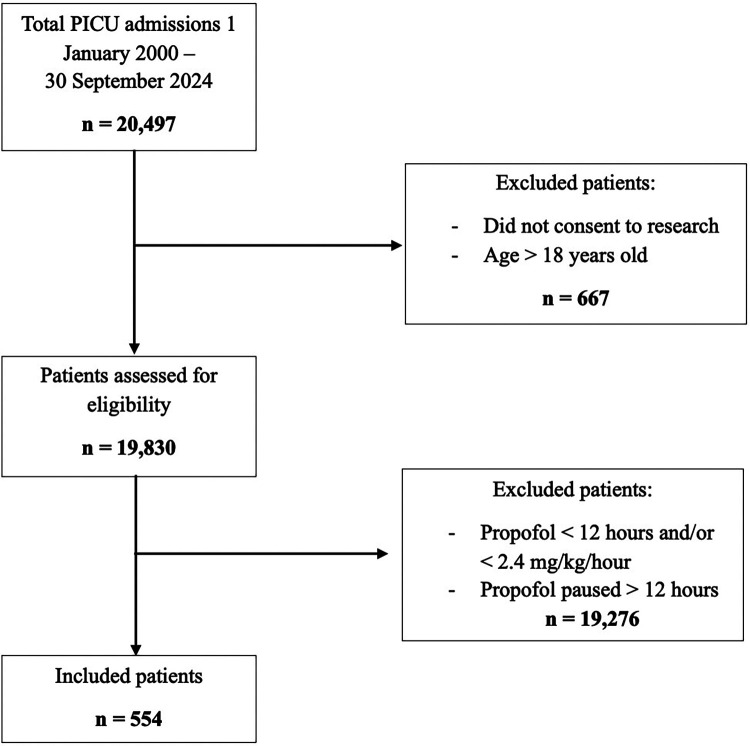
Flow diagram illustrating study population selection.

Patients were included if they received continuous propofol infusions for non-procedural sedation for duration ≥12 h and/or dose ≥2.4 mg/kg/hour (40 mcg/kg/min). Patients were excluded if aged ≥18 years, status post cardiac surgery, received propofol for <12 h, or experienced a continuous pause >12 h during infusion. Per Minnesota law, patients who declined the use of their medical records for research purposes were not included. The full inclusion process is summarized in Figure 1.

### Data collection

Patient identification and data extraction used the Multidisciplinary Epidemiology and Translational Research in Intensive Care Data Mart (METRIC DataMart), an electronic database housing comprehensive ICU data, supplemented by manual chart review (10).

We retrospectively extracted data from the patient's electronic medical record, including demographics, admission diagnosis, propofol rates and duration, acid-base status, heart rate variation, and concurrent use of other analgesic and sedative medications (fentanyl, midazolam, chloral hydrate, dexmedetomidine, ketamine, hydromorphone, morphine, and lorazepam). Mechanical ventilation assessment was followed by pulse oximetry and end-tidal CO_2_.

To evaluate PRIS-related characteristics, we collected the following information during propofol infusion: median heart rate, lowest heart rate, presence of bradycardia (defined as heart rate <60 bpm), incidence of metabolic acidosis (defined as lactate ≥3 mmol/L and/or base deficit ≥ −10 mEq/L), and use of vasopressors, chronotropes or inotropes.

### Definition of PRIS

To maintain consistency with prior literature, PRIS was defined as sudden or relatively sudden, treatment-resistant progressive bradycardia; and at least one of the following (1, 8):

- Metabolic acidosis (arterial base deficit ≥10 mEq/L)
- Lipemic plasma (serum triglycerides ≥2.3 mmol/L)
- Muscle involvement (rhabdomyolysis and/or myoglobinuria)
- Hepatomegaly on clinical examination or fatty liver infiltration on autopsy

Bradycardia was considered PRIS-related only if accompanied by signs of hemodynamic instability necessitating chest compressions and/or use of chronotropic, inotropic or vasopressor drugs (such as atropine or epinephrine) (11).

### Statistical analysis

The true incidence of PRIS in critically ill infants and children remains uncertain largely due to inconsistent diagnostic criteria and small sample sizes. However, adult critical care literature suggests an incidence of approximately 1.1% (12). To minimize the risk of Type II error, this study was powered based on an expected PRIS incidence of 1.1%, with a calculated sample size of 490 needed to detect a clinically significant difference (*α* = 0.05, margin of error= 1%). With a final cohort of 554 patients, the study is adequately powered.

The primary outcome of interest was the incidence of PRIS (as defined above). Secondary outcomes included intensive care unit (ICU) length of stay and survival to hospital discharge. Categorical variables were summarized using frequencies and percentages. Continuous variables were reported as medians with interquartile ranges (IQR). Comparisons across categorized age groups (<6 months, 6–12 months, 1–5 years, and 6–17 years) and diagnostic type (medical vs. surgical) were performed using the chi-square test for homogeneity and the Kruskal–Wallis test, as appropriate. Statistical significance was defined as *p* < 0.05.

## Results

### Study population

A total of 554 patients met the inclusion criteria. The median age was 5.5 (IQR 1.5–12.1) years. Of all patients, 59% were male and 98% were mechanically ventilated. There was representation in all categorized admitting diagnoses: 42% medical and 58% surgical. Patient demographic characteristics, categorized by age group (<6 months, 6–12 months, 1–5 years, and 6–17 years), are summarized in Table 1.

**Table 1:** Patient demographics by age group.

Variables	Overall (*n* = 554)	<6 months (*n* = 40)	6–12months (*n* = 54)	1–5 years (*n* = 173)	6–17 years (*n* = 287)	*p*-value
Age in years (median, IQR)	5.5 (1.5–12.1)	0.3 (0.2–0.4)	0.9 (0.7–1.0)	2.1 (1.5–3.2)	11.8 (8.2–14.6)	<0.001
Male sex (*n*, %)	329 (59%)	30 (75%)	35 (65%)	97 (56%)	167 (58%)	0.68
Mechanical ventilation (*n*, %)	542 (98%)	40 (100%)	53 (98%)	170 (98%)	279 (97%)	0.99
Medical diagnoses (*n*, %)	235 (42.4%)	24 (60%)	32 (59%)	71 (41%)	108 (37.6%)	
Medical respiratory	132 (24%)	21 (53%)	23 (43%)	40 (23%)	48 (17%)	<0.001
Medical neurological	43 (8%)	2 (5%)	4 (7%)	17 (10%)	20 (7%)	0.81
Medical metabolic	24 (5%)	0 (0%)	2 (4%)	3 (2%)	19 (7%)	0.09
Medical infectious	19 (3%)	0 (0%)	2 (4%)	4 (2%)	13 (5%)	0.55
Medical cardiac	17 (3%)	1 (3%)	1 (2%)	7 (4%)	8 (3%)	0.92
Surgical diagnoses (*n*, %)	319 (57.6%)	16 (40%)	22 (41%)	102 (59%)	179 (62.4%)	
Surgical otolaryngological	113 (20%)	13 (33%)	16 (30%)	57 (33%)	27 (9%)	<0.001
Surgical orthopedic	58 (10%)	1 (3%)	2 (4%)	6 (3%)	49 (17%)	<0.001
Surgical neurological	52 (10%)	1 (3%)	1 (2%)	13 (8%)	37 (13%)	0.05
Surgical trauma	49 (9%)	0 (0%)	1 (2%)	10 (6%)	38 (13%)	0.006
Surgical general	47 (8%)	1 (3%)	2 (4%)	16 (9%)	28 (10%)	0.43

IQR, interquartile range; *n*, number; %, percentage.

Percentages may exceed 100% due to rounding.

We classified our patients based on physiological changes in relation to propofol metabolism. Propofol's metabolism in pediatrics is influenced by age and body weight. The pharmacokinetics of the pediatric population exhibit a larger central compartment, increased metabolic clearance, and larger volumes of distribution compared to adults, resulting in increased clearance and a greater volume of distribution (13). These characteristics result in higher induction and maintenance doses in children compared to adults. Propofol is metabolized in the liver, and infants have immature liver activity that matures as they grow older, reaching maturity at around 30 weeks, hence our distinction between 0 and 6 months, and 6-12 months (14). Additionally, there have been three periods described associated with propofol pharmacokinetics: Neonates, where clearance is only 10% in those born premature and 38% in those born at term, and 90% in adults (15). Infants have increased volume of distribution in the first year of life and faster clearance, and it also varies in children at 2 years of age. In prepubertal children, volumes are nearly twice greater compared to adults (15).

All age groups were similar with respect to patient sex, invasive ventilator use and the proportion of medical vs. surgical diagnoses. Not unexpectedly, statistically significant differences were observed across age groups for medical respiratory (*p* < 0.001), surgical otolaryngological (*p* < 0.001), surgical trauma (*p* < 0.001) and surgical orthopedic diagnoses (*p* = 0.006).

Laboratory data was not available for all patients due to their lack of an arterial line. These patients had hemodynamic stability, were not on high ventilator settings, or had short-term use of mechanical ventilation. Patients were continuously monitored, and the development of bradycardia would lead to a physical exam, hemodynamic assessment, and laboratory work-up, including a blood gas, basic metabolic panel for electrolytes and base deficit, and a complete blood count with inflammatory markers if the patient developed hypotension.

### Propofol infusion characteristics

The characteristics of propofol infusion, categorized by age group, are summarized in Table 2. The median infusion rate was 3.0 (IQR 1.7–4.2) mg/kg/hour [50 (28–70) mcg/kg/min], with minimum and maximum doses of 0.11 and 54 mg/kg/hour (1.8 and 900 mcg/kg/min), respectively. The median infusion duration was 22.8 (IQR 16.4–42.4) hours, with minimum and maximum durations of 12.0 and 203.8 h, respectively. The median cumulative dose (including additional 1 mg/kg boluses) was 73.9 (IQR 36.4–130.4) mg/kg. Infusion rates were statistically significant across age groups (*p* < 0.001), with patients aged 1–5 years receiving the highest median infusion rate (3.6 mg/kg/hour or 60 mcg/kg/min) compared to other age groups, followed by 6–12 months (3.1 mg/kg/hour or 52 mcg/kg/min), 6–17 years (2.5 mg/kg/hour or 42 mcg/kg/min), and <6 months (2.4 mg/kg/hour or 40 mcg/kg/min).

**Table 2:** Propofol infusion characteristics by Age group.

Variables	Overall (*n* = 554)	<6 months (*n* = 40)	6–12 months (*n* = 54)	1–5 years (*n* = 173)	6–17 years (*n* = 287)	*p*-value
Infusion rate mg/kg/hour (median, IQR)	3.0 (1.7–4.2)	2.4 (1.5–4.4)	3.1 (2.0–4.2)	3.6 (2.1–4.7)	2.5 (1.4–3.7)	<0.001
Infusion duration in hours (median, IQR)	22.8 (16.4–42.4)	25.8 (18.4–43.1)	20.1 (15.3–29.0)	22.6 (16.5–40.5)	23.5 (16.4–46.0)	0.24
Cumulative dose mg/kg (median, IQR)	73.9 (36.4–130.4)	66.7 (33.6–129.2)	72.3 (46.2–116.2)	83.3 (41.0–158.3)	67.8 (34.4–128.1)	0.07

IQR, interquartile range; *n,* number; mg, milligrams; kg, kilograms.

It is important to note that 156 patients (28%) received median doses >4 mg/kg/hour (67 mcg/kg/min), and 12 patients (2%) received doses >8 mg/kg/hour (133 mcg/kg/min). Additionally, 119 patients (21%) received propofol infusions for durations >48 h, and 40 patients (7%) exceeded 96 h.

**Figure 2:**
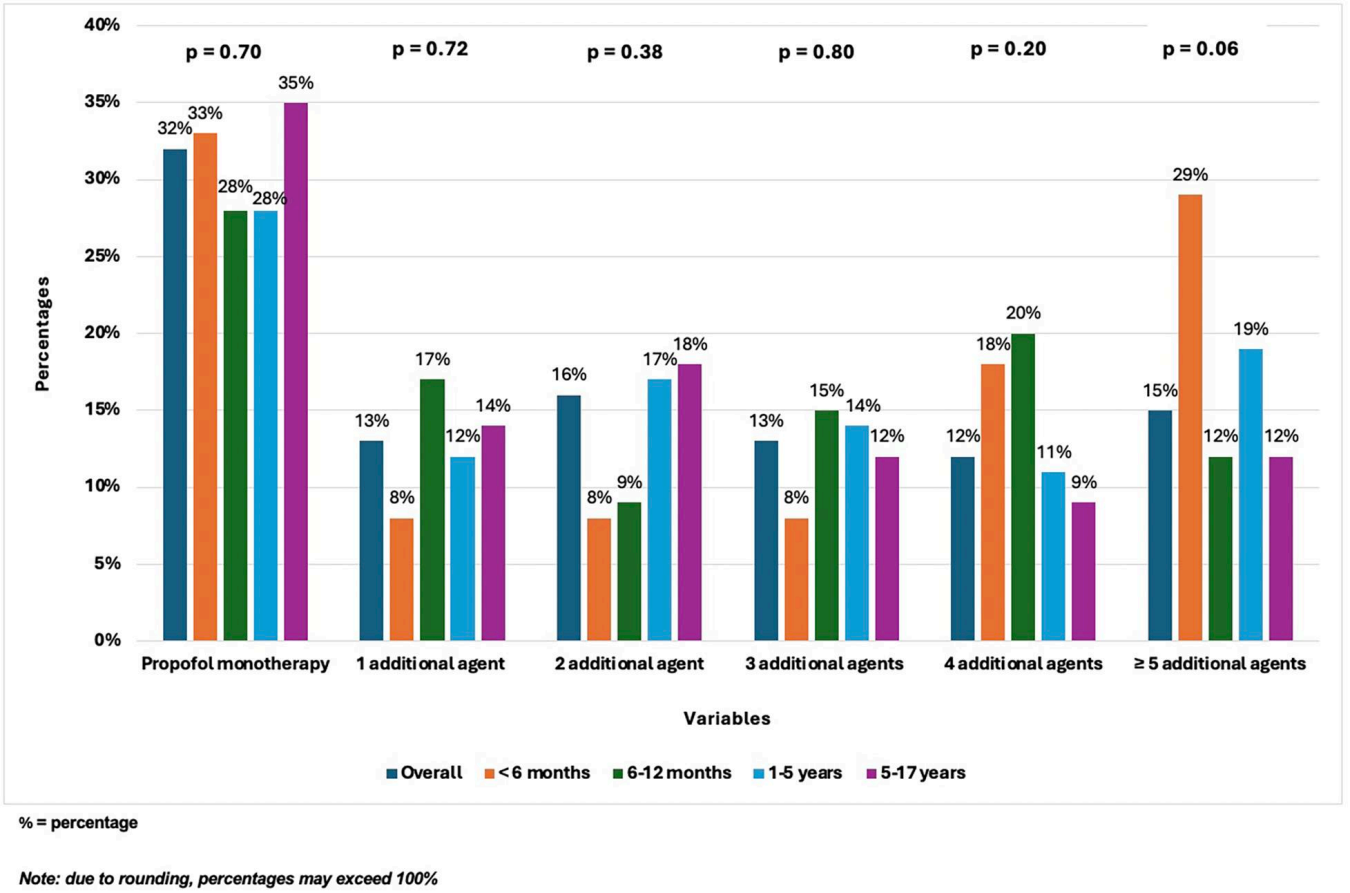
Concomitant sedation during propofol infusions.

### Concomitant sedation

The highest proportion of patients in each age group were maintained on propofol monotherapy for non-procedural sedation (28%–35%), mostly in preparation for extubation. However, as shown in Figure 2, many required additional agents to achieve effective sedation: 13% received 1 additional agent, 16% 2 additional agents, 13% 3 additional agents, 12% 4 additional agents, 15% ≥5 additional agents. Although the use of concomitant sedation was not statistically significant across age groups, a trend toward increased use in younger patients, particularly those <6 months, was observed (18% 4 additional agents, 29% ≥5 additional agents). The most commonly used concomitant agents included fentanyl (57%), dexmedetomidine (50%), midazolam (37%), lorazepam (21%), ketamine (20%), hydromorphone (14%), chloral hydrate (11%), and morphine (6%).

**Figure 3:**
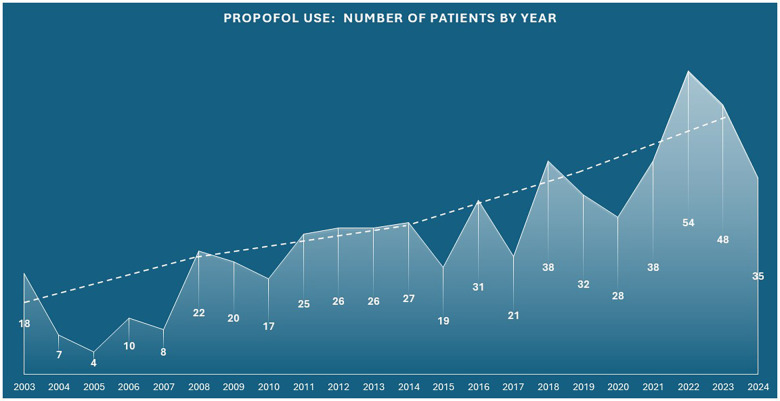
Annual PICU number of patients receiving Propofol from 2003 through 2024..

Additionally, Figure 3 illustrates the annual number of PICU patients receiving continuous propofol infusions from 2000 to 2024 (per inclusion criteria), demonstrating a steady increase in use over time.

### Outcome measures

Table 3 summarizes the outcome data by age group. There was one observed case of PRIS in the cohort. This occurred in a 15-year-old patient with diffuse axonal injury on propofol monotherapy. The patient received a median infusion rate of 1.0 mg/kg/hour (17 mcg/kg/min) over 42.1 h. Epinephrine was initiated due to hypotension, and the patient was found to have metabolic acidosis (lactate 6.1 mmol/L, base deficit −9 mEq/L), which resolved promptly after discontinuation of propofol (lactate 1.1 mmol/L). The median and lowest heart rates were 73 and 51 bpm, respectively. The patient did not require chest compressions and did not progress to asystole. The patient survived to hospital discharge.

**Table 3:** Outcome measures by Age group.

Variables	Overall (*n* = 554)	<6 months (*n* = 40)	6–12 months (*n* = 54)	1–5 years (*n* = 173)	6–17 years (*n* = 287)	*p*-value
Lowest heart rate during infusion in bpm (median, IQR)	75.0 (59.0–90.0)	86.5 (78.8–99.0)	89.5 (73.3–105.5)	78.0 (66.0–93.0)	67.0 (55.0–82.0)	<0.001
Bradycardia during infusion (*n*, %)	146 (26%)	3 (8%)	7 (13%)	33 (19%)	103 (36%)	<0.001
Highest lactate during infusion mmol/L (median, IQR)	1.3 (0.9–2.0)	1.1 (0.9–1.6)	0.9 (0.7–0.9)	1.3 (0.9–1.9)	1.4 (1.0–2.2)	0.06
Lowest base deficit during infusion mEq/L (median, IQR)	−3.0 (−5 to −1)	−3.0 (−5 to 5.3)	−2.0 (−4 to 0.3)	−4.0 (−6.0 to 0.0)	−3.0 (−5 to −1)	0.11
Acidosis during infusion (*n*, %)	19 (7%)	1 (6%)^a^	0 (0%)^a^	8 (10%)^a^	10 (6%)^a^	0.61
ICU length of stay in days (median, IQR)	4.7 (2.0–10.4)	7.6 (2.9–14.3)	4.9 (2.0–9.7)	4.9 (2.2–11.8)	4.1 (2.0–8.9)	0.07
Survival to hospital discharge (*n*, %)	528 (95%)	37 (93%)	52 (96%)	164 (95%)	275 (96%)	0.99
Incidence of PRIS (*n*, %)	1 (0.2%)	0 (0%)	0 (0%)	0 (0%)	1 (0.4%)	0.82

bpm, beats/minute; IQR, interquartile range; *n*, number; %, percentage; ICU, intensive care unit; PRIS, propofol-related infusion syndrome.

a indicates varying percentages due to missing data.

The median length of stay in the ICU was 4.7 (IQR 2.0–10.4) days, and 528 (95%) of patients survived until hospital discharge.

Bradycardia occurred in 146 (26%) patients and was statistically significant across age groups. Patients aged 6–17 years had the lowest median heart rate (67 bpm, *p* < 0.001) and the highest incidence of bradycardia [103 patients (36%), *p* < 0.001] during infusion. Among those with bradycardia, 5 patients (1%) required intervention: all required chronotropic agents (atropine and/or epinephrine) and three required chest compressions. Manual chart review revealed that only one case had clinical features concerning for PRIS: including metabolic acidosis, rhabdomyolysis, myoglobinuria, elevated triglycerides, and hepatomegaly. Three of these five cases were attributed to respiratory causes (laryngospasm, hydrothorax, endotracheal tube dislodgement) and continuous propofol infusion was maintained post-event in four cases.

Metabolic acidosis was observed in 19 of the 264 (7%) patients with recorded blood gases. The median of the highest lactate values was 1.3 mmol/L, and the median of the lowest base deficits was −3.0 mEq/L. Although the overall acidosis incidence did not differ significantly by age group, patients aged 1–5 years had the lowest median base deficit (−4.0 mEq/L, *p* < 0.001) and the highest incidence of acidosis [8 patients (10%), *p* = 0.04].

When two characteristics were present, such as bradycardia and acidosis, the propofol infusion was decreased, and a different sedative was added. We observed this association in 13% of our population.

### Diagnostic type

As summarized in Table 4, patients with a medical diagnosis were significantly younger than those with surgical diagnosis (3.6 vs. 7.2 years, *p* = 0.002). There were no differences in sex, mechanical ventilation, infusion rate, cumulative dose, or mortality between the two groups. Interestingly, patients with medical diagnoses had longer ICU lengths of stay (5.0 vs. 4.0 days, *p* < 0.02) and longer propofol infusion durations (24.9 vs. 21.5 h, *p* = 0.01). The incidence of acidosis and bradycardia was similar between groups. One surgical trauma patient met the criteria for PRIS.

**Table 4:** Patient demographics and outcome measures by diagnostic type.

Variables	Overall (*n* = 554)	Medical (*n* = 235)	Surgical (*n* = 319)	*p*-value
Age in years (median, IQR)	5.5 (1.5–12.1)	3.6 (1.1–11.3)	7.2 (1.9–12.7)	0.002
Male sex (*n*, %)	329 (59%)	147 (63%)	182 (57%)	0.41
Mechanical ventilation (*n*, %)	542 (98%)	229 (97%)	313 (98%)	0.94
Infusion rate mg/kg/hour (median, IQR)	3.0 (1.7–4.2)	3.0 (1.7–4.2)	3.0 (1.7–4.2)	0.97
Infusion duration in hours (median, IQR)	22.8 (16.4–42.4)	24.9 (16.7–49.2)	21.5 (16.0–39.2)	0.01
Cumulative dose mg/kg (median, IQR)	73.9 (36.4–130.4)	76.6 (40.0–158.0)	72.2 (35.4–118.6)	0.11
Propofol monotherapy (*n*, %)	177 (32%)	68 (29%)	109 (34%)	0.28
Lowest heart rate during infusion in bpm (median, IQR)	75.0 (44–106)	76.0 (41–111)	74.0 (45–103)	0.16
Bradycardia during infusion (*n*, %)	146 (26%)	61 (26%)	85 (27%)	0.88
Highest lactate during infusion mmol/L (median, IQR)	1.3 (0.9–2.0)	1.3 (0.9–1.9)	1.3 (0.9–2.2)	0.84
Lowest base deficit during infusion mEq/L (median, IQR)	−3.0 (−5.0 to −1.0)	−3.0 (−5.0 to 0.0)	−2.0 (−5.0 to −1.0)	0.80
Acidosis during infusion (*n*, %)	19 (7%)^a^	9 (8%)^a^	10 (6%)^a^	0.66
ICU length of stay in days (median, IQR)	4.7 (2.0–10.4)	5.0 (2.2–12.8)	4.0 (2.0–8.8)	0.02
Survival to hospital discharge (*n*, %)	528 (95%)	215 (91%)	313 (98%)	0.43
Incidence of PRIS (*n*, %)	1 (0.2%)	0 (0%)	1 (0.3%)	0.39

bpm, beats per minute; IQR, interquartile range; *n*, number; %, percentage; ICU, intensive care unit; PRIS, propofol-related infusion syndrome.

a indicates varying percentages due to missing data.

Table 5 presents the variables collected in our patient population associated with PRIS.

**Table 5:** Patients with PRIS characteristics.

Age	0-≤6 months	6–12 months	1–5 years	6–17 years	Total
Total number of patients	40	54	173	287	554
Patient number with available arterial blood gas	15	20	78	151	264
Bradycardia	3	7	33	103	146
Acidosis	1	0	8	10	19
Lactic acidemia	1	0	6	11	17
Myoglobinuria	0	0	0	1	1

### Mortality

As summarized in Table 6, no deaths were attributed to PRIS. Among the 26 (4.7%) patients who did not survive to hospital discharge, the median age was 4.6 (IQR 1.2–10.7) years, with a median ICU stay of 8.8 (IQR 3.4–19.5) days. Mechanical ventilation was required in 89%. The median propofol infusion rate was 3.0 (IQR 1.6–3.8) mg/kg/hour [50 (IQR 27–63) mcg/kg/min], with a median duration of 33.6 (IQR 21.0–61.3) hours. Among these patients, there was a higher incidence of bradycardia [8 patients (30%)] and acidosis [8 out of 18 patients with record blood gases (44%)]. All deaths were attributed to underlying disease processes, not to propofol administration.

**Table 6:** Patient mortality.

Case number	Age (years)	Cause of death	Median infusion rate (mg/kg/hour)	Infusion duration (hours)	Bradycardia during infusion	Acidosis during infusion
1	0.2	Anoxic brain injury due to cardiac arrest	7.50	27.92	No	N/A
2	0.3	Respiratory failure due to pulmonary hypertension	1.50	35.75	No	Yes
3	0.3	Multiorgan failure due to sepsis	3.60	91.72	No	No
4	0.6	Respiratory failure due to pulmonary hypertension	1.20	111.25	No	N/A
5	1.0	Anoxic brain injury due to drowning	0.11	20.94	No	No
6	1.1	Cerebral hemorrhage due to non-accidental trauma	0.63	14.36	No	N/A
7	1.1	Respiratory failure due to necrotizing pneumonia	4.20	16.88	No	No
8	1.3	Anoxic brain injury due to cardiac arrest	1.80	17.18	No	N/A
9	1.3	Respiratory failure due to post-transplant lymphoproliferative disease	3.60	119.33	Yes	No
10	1.4	Multiorgan failure due to sepsis	3.90	49.75	Yes	N/A
11	2.6	Cerebral hemorrhage due to a gunshot wound	4.50	52.32	No	No
12	4.3	Respiratory failure due to metastatic rhabdomyosarcoma	3.90	73.30	Yes	Yes
13	4.5	Brain herniation due to metastatic medulloblastoma	3.64	28.16	No	N/A
14	4.6	Traumatic brain injury due to a motor vehicle accident	3.00	28.05	Yes	Yes
15	5.6	Traumatic brain injury due to a motor vehicle accident	1.26	147.31	No	Yes
16	7.9	Brain herniation due to grade IV glioblastoma	4.50	64.32	Yes	N/A
17	8.0	Respiratory failure due to pulmonary hemorrhage	3.00	21.30	No	No
18	9.9	Respiratory failure due to idiopathic pneumonia syndrome	3.60	36.10	No	No
19	10.5	Brain herniation due to bacterial meningitis	6.00	18.97	No	Yes
20	10.7	Anoxic brain injury due to respiratory failure	3.00	16.50	No	N/A
21	11.7	Anoxic brain injury due to drowning	1.80	38.98	No	No
22	13.1	Anoxic brain injury due to hanging	0.50	34.41	No	No
23	13.1	Anoxic brain injury due to hanging	2.27	29.43	No	Yes
24	13.4	Traumatic brain injury due to a motor vehicle accident	0.25	29.08	Yes	Yes
25	16.5	Multiorgan failure due to sepsis	1.80	163.02	Yes	No
26	16.8	Fulminant liver failure due to acetaminophen ingestion	3.40	32.85	Yes	Yes

mg, milligrams; kg, kilogram; bpm, beats/minute; mmol/L, millimoles per liter.

N/A indicates missing data.

## Discussion

This study is a large-scale evaluation of continuous propofol administration for non-procedural sedation within a non-cardiac medical/surgical PICU population. It characterizes the clinical course, adverse events, and outcomes associated with propofol sedation in a general PICU population. Among the 554 children included, the incidence of PRIS was low (0.2%), even when propofol was administered at doses and durations historically considered high-risk. Bradycardia occurred in 26% of patients; however, only 1% required medical intervention, and only one of these events was attributed to PRIS. Continuous propofol infusion was maintained post-event in four of the five events.

PRIS is a challenging clinical diagnosis supported by laboratory results. There is a lack of a specific biomarker to determine the presence or absence of PRIS. Since PRIS is primarily a clinical diagnosis (characterized by hemodynamic instability with bradycardia and hypotension) and associated with significant laboratory findings (metabolic acidosis and elevated lactate), it is valuable to consider PRIS in the differential diagnosis of a specific critically ill patient. Sometimes, urine color changes can lead to the suspicion of PRIS, such as red urine for rhabdomyolysis or the “green urine” originally described in lethal cases of propofol.

Not all mechanically ventilated pediatric patients require an arterial line. Bedside providers rely on pulse oxymetry (oxygenation) and end-tidal CO2 (ventilation). Arterial lines, particularly in small pediatric patients, can be challenging to initiate and may require multiple attempts before a successful placement. Specific recommendations for arterial line placement include hemodynamic instability (shock), continuous and accurate blood pressure monitoring while using vasoactive infusions, the need for frequent blood gases in severe respiratory failure, rapidly changing ventilator settings, and high ventilatory support requirements. A recent survey among 377 pediatric critical care practitioners across 93 institutions in the USA showed substantial variability in clinical practice despite some practice guidelines in existence (16). Arterial lines in our practice are not needed if the patient meets the following clinical criteria: hemodynamically stable, not on vasoactive medications, no need for frequent arterial blood gases, low mechanical ventilator settings or short duration of mechanical ventilation. In the patient population included in this study, daily blood gas is not a common practice unless hemodynamic instability develops or if the patient develops bradycardia along with hypotension, who does not respond to a decrease in propofol infusion.

Notably, the single case of PRIS occurred in a patient who received propofol at a rate (1.0 mg/kg/hour or 16 mcg/kg/min) and duration (42 h) within current recommendations. Although the exact mechanism behind the development of PRIS is unknown, current theories suggest propofol disrupts mitochondrial function, particularly under conditions of physiological stress such as trauma, surgery, or sepsis—an effect resembling inherited mitochondrial disorders (3, 17–19). Pediatric studies have identified several risk factors for PRIS, including high cumulative doses, long durations, young age, critical illness, as well as concomitant catecholamine or corticosteroid use (1–3). However, rare cases have occurred with lower doses and shorter durations in patients with underlying genetic mitochondrial defects, suggesting that individual susceptibility may also play a role in PRIS pathogenesis (3, 20, 21).

The 2022 Society of Critical Care Medicine (SCCM) clinical practice guidelines on the Prevention and Management of Pain, Agitation, Neuromuscular Blockade, and Delirium in Critically Ill Pediatric Patients (PANDEM) recommended dexmedetomidine as the preferred sedation agent for pediatric patients requiring mechanical ventilation (22). However, the guidelines also support propofol as a safe alternative when used at doses <4 mg/kg/hour (66 mcg/kg/min) for durations <48 h, to minimize the risk of PRIS (22). Propofol's pharmacologic profile—rapid onset, fast recovery, relatively low accumulation, and minimal post-sedation nausea—make it an excellent option for pediatric sedation in select scenarios (15). Propofol use in the PICU has been associated with earlier separation from mechanical ventilation compared to midazolam (23). In adult ICU populations, propofol has been linked to lower mortality, reduced ventilator days, and shorter ICU stays compared to midazolam (24). Additionally, in pediatric patients undergoing sedated MRI, propofol was associated with superior outcomes in emergence time, parental satisfaction, procedural timeliness, and postoperative behavior compared to dexmedetomidine (25).

Despite these advantages, propofol use remains highly variable across institutions, nationally and internationally. Clinicians generally limit its use to specific situations, shorter durations, lower doses, and older age groups (26). A 1997 survey of SCCM members found that only 22% of respondents used propofol frequently in pediatric patients, typically for brief periods, with clinician preference, experience, and drug duration being key factors in sedative selection (27). More recent surveys show increasing use. A 2002 survey of Australian and New Zealand PICUs found 39% of respondents considered propofol appropriate for long-term sedation of mechanically ventilated pediatric patients (5). Similarly, a 2012 national survey in Germany found 79% of PICUs endorsed propofol use in children under 16 years, 98% for bolus application and 78% for infusion ≥3 h (5). Finally, a 2016 survey of Israeli PICU physicians found propofol to be widely accepted, with 40% administering it without specifying an upper dose limit (6).

Historically, propofol doses >4 mg/kg/hour (66 mcg/kg/min) and durations >48 h have been considered high risk for PRIS in the pediatric population (1). In our study, while the median dose and median duration of 22.8 h fell within those ranges, there were a number of patients who received higher exposures. These dosing ranges and durations significantly surpass those typically reported in the literature (8). The low incidence of PRIS and absence of propofol-related mortality in this high-risk cohort suggests an opportunity to reassess historical dosing thresholds and provides an opportunity to safely use propofol as a continuous agent in the pediatric critical care unit.

### Limitations

Although our study suggests a reassuring safety profile for propofol in the modern PICU era, several limitations should be considered. First, as a single-center, retrospective observational study, our design lacks randomization and a control group, which limits the ability to make causal inferences. Second, clinical features of critical illness (hemodynamic instability, metabolic and lactic acidosis, bradycardia and cardiac rhythm abnormalities) can be difficult to differentiate from propofol-related adverse events, making it challenging to prove these clinical events are related to propofol or the patient's critical condition. Third, 50% of patients received dexmedetomidine concurrently, and our population included neurosurgical and trauma patients with raised intracranial pressure, both of which may have contributed to the incidence of bradycardia. Fourth, consistent identification of PRIS was limited by missing data; diagnostic markers such as lipemia and rhabdomyolysis are not routinely checked unless a high index of suspicion is present. Hepatomegaly assessment is part of the daily physical examination, and blood gases to assess metabolic acidosis are not routinely checked in critical care. However, considering that most of our patients did not require an arterial line and blood gases were not routinely obtained, the clinical condition of the patient led the medical team to pursue further investigation if the patient were to become hemodynamically unstable (hypotension, bradycardia) or show changes in urine color (indicating rhabdomyolysis). Fifth, although our overall sample size exceeded the threshold needed to detect a PRIS incidence of 1.1%, approximately 75% of patients received propofol infusions within PANDEM-recommended dosing and duration ranges. This resulted in a smaller number of patients exposed to doses >4 mg/kg/hour or durations >48 h, thereby limiting the power to evaluate PRIS risk specifically within the highest-exposure subgroup. Finally, while our findings are likely reproducible in tertiary academic PICUs, generalizability may be limited in centers with mixed units, particularly those with higher-risk cardiac surgical patients who have unique hemodynamic vulnerabilities, often require prolonged sedation, and may lack readily available data. Additionally, institutional differences in provider training, sedation protocols, and PRIS screening practices may further limit broader applicability.

## Conclusions

In this single-center, retrospective observational study on sedation management in a non-cardiac medical/surgical PICU, continuous propofol infusion was associated with a low incidence of PRIS, even when both the dose and duration exceeded literature-based recommendations. One case of PRIS was observed, and there were no inpatient deaths attributed to propofol use. This study provides important safety data for a broad PICU population, excluding the confounding cardiac surgical cohort. Given the low incidence of PRIS in this cohort, these findings support the cautious use of propofol for non-procedural sedation in critically ill infants and children without congenital or acquired heart disease.

## Data Availability

The raw data supporting the conclusions of this article will be made available by the authors, without undue reservation.
